# The Effect of Tumor Location and Extension on Survival in Patients with Sinonasal Mucosal Melanoma: A Systematic Review and Meta-Analysis

**DOI:** 10.3390/cancers17233757

**Published:** 2025-11-25

**Authors:** Fan Yang, Marina Ruiz Cifuentes, Peter Horvatovich, Victor Guryev, Eszter Baltás, Gilles F H Diercks, Alienke van Pijkeren, Inge Wegner, Gyorgy B Halmos

**Affiliations:** 1 Department of Otorhinolaryngology, Head and Neck Surgery, University Medical Center Groningen, University of Groningen, 9700 RB Groningen, The Netherlands; m.ruiz.cifuentes@umcg.nl (M.R.C.); i.wegner@umcg.nl (I.W.); g.b.halmos@umcg.nl (G.B.H.); 2 Department of Analytical Biochemistry, Faculty of Science and Engineering, University of Groningen, 9713 AV Groningen, The Netherlands; p.l.horvatovich@rug.nl (P.H.); a.van.pijkeren@rug.nl (A.v.P.); 3 European Research Institute for the Biology of Ageing, University Medical Center Groningen, University of Groningen, 9713 AV Groningen, The Netherlands; v.guryev@umcg.nl; 4 Department of Dermatology and Allergology, University of Szeged, 6720 Szeged, Hungary; baltas.eszter@med.u-szeged.hu; 5 Department of Pathology and Medical Biology, University Medical Center Groningen, University of Groningen, 9700 RB Groningen, The Netherlands; g.f.h.diercks@umcg.nl

**Keywords:** sinonasal mucosal melanoma, tumor location, tumor extension, meta-analysis, overall survival, prognosis

## Abstract

Background/Objectives: SNMM is a rare and aggressive malignancy with a poor prognosis. The current staging systems fail to adequately stratify patient risk. This study aimed to evaluate the prognostic impact of tumor location and extension on overall survival (OS) in SNMM. Methods: A systematic literature search of Medline, Web of Science, and Embase was performed to identify studies assessing the prognostic significance of tumor location and extension. Study quality was evaluated using the Quality in Prognosis Studies (QUIPS-2) tool. Meta-analyses were conducted to calculate pooled hazard ratios (HRs) with 95% confidence intervals (CIs). Eligible studies included primary SNMM reporting tumor location/extension and survival; observational designs (case series ≥ 5 patients) were eligible with no language restrictions. Searches covered MEDLINE, Web of Science, and Embase and were last updated on 10 Jan 2025; reference lists were also screened. Results: Thirty-four studies were included in the systematic review, of which ten met criteria for meta-analysis. Tumors located in the paranasal sinuses (HR = 2.89, 95% CI: 1.63–5.14) and those with orbital involvement (HR = 1.92, 95% CI: 1.34–2.73) were associated with significantly poorer OS. Maxillary and ethmoid sinus involvement showed no statistically significant difference compared with nasal cavity tumors. Conclusions: Tumor location and extension are significant prognostic indicators in SNMM. Patients with paranasal sinus tumors or orbital invasion have worse outcomes, supporting inclusion of these factors in future staging systems for better clinical decision-making. Limitations include the observational nature of the evidence, heterogeneity across definitions and analyses, and underpowered publication-bias tests; certainty of evidence was not formally graded.

## Simple Summary

Sinonasal mucosal melanoma (SNMM) is a rare and aggressive cancer arising in the nasal cavity or paranasal sinuses. This study systematically reviewed and analyzed previously published data to determine whether the tumor’s location and extent of invasion influence patient survival. The findings reveal that tumors originating in paranasal sinuses and those extending into orbit are associated with poorer outcomes. These results highlight the importance of anatomical factors in prognosis and suggest that future staging systems should include tumor location and extension to improve risk stratification and guide treatment decisions.

## 1. Introduction

Sinonasal mucosal melanoma (SNMM) is a rare and aggressive malignancy with poor prognosis, accounting for 0.7–1% of all melanomas and 60–70% of head and neck mucosal melanoma (HNMM) [1]. The 5-year overall survival (OS) of SNMM is 36.6% in Europe, 28.1% in Asia and 40.5% in the US [1]. Surgical resection remains the primary treatment for localized SNMM [2,3], but due to the complex anatomy of the region, achieving negative margins is often challenging. Therefore, postoperative radiotherapy (PORT) and immune checkpoint inhibitors (ICIs) are often applied as adjuvant treatment; however, literature data on their efficacy are controversial [4,5,6]. Mono and combination ICIs appear to be significantly less effective in SNMM than those in cutaneous melanoma [7] in both metastatic and neoadjuvant settings, suggesting a distinct genetic background and behavior. The American Joint Committee on Cancer (AJCC) 8th Tumor Node Metastasis (TNM) classification system is widely used for the staging of SNMM [8]; however, it classifies the primary SNMM into T3 (tumor confined to the mucosa and immediately underlying soft tissue) and T4 (invasion beyond the submucosa into deeper structures) categories and cannot accurately perform risk-based classification. Moreover, most patients with SNMM present with T4 tumors, as T3 tumors are usually asymptomatic. In contrast, the sinonasal-specific TNM classification for non-melanoma cancers provides a more detailed stratification with T1–T4 stages, considering factors such as tumor size and local invasion. It has been suggested that integrating these two classification systems may improve risk stratification for SNMM patients [9]. Some researchers have found that tumor location and extension may be predictive of different survival outcomes in SNMM patients [9,10,11,12]. Although a staging system for SNMM exists, a comprehensive analysis comparing tumor location and extension with patient prognosis has not yet been conducted. To date, no meta-analysis has systematically addressed this issue. Therefore, this study aims to fill this gap by synthesizing available evidence to identify prognostic factors that may guide refinement of future staging systems.

## 2. Materials and Methods

### 2.1. Reporting Guideline, Registration, and Ethics

**Figure 1:**
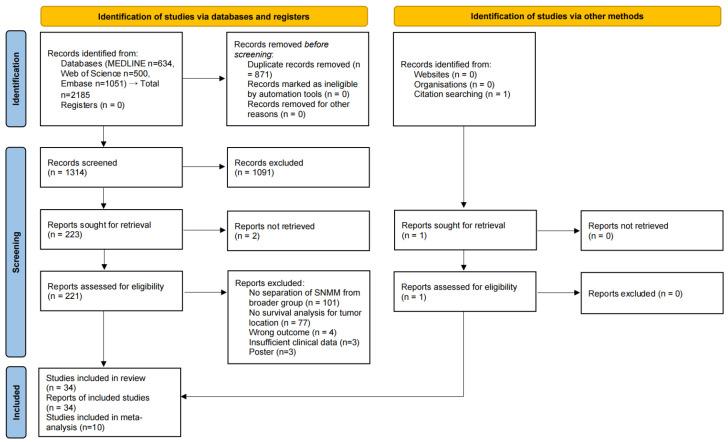
PRISMA 2020 flow diagram of the study selection process.

This systematic review and meta-analysis followed PRISMA 2020. The completed PRISMA checklist and flow diagram are provided in the Supplementary Materials and Figure 1. The protocol was prospectively registered in PROSPERO (CRD42025634023). Search reporting adheres to PRISMA-S; full, reproducible strategies for each database are given in Appendix A.1. Ethical approval was not required because only previously published data were analyzed.

### 2.2. Definitions

In this study, the primary tumor site was categorized based on anatomical location, where the tumor was most likely originating from. For tumors involving both the nasal cavity and paranasal sinuses, we followed the original studies’ classification. When unclear, lesions were categorized as ‘overlapping’ or ‘not otherwise specified (NOS)’, as reported in the individual studies. Tumor extension (different site involvement) refers to the spread within the sinonasal region, including the nasal cavity and the different paranasal sinuses. Tumor invasion was defined as infiltration beyond the mucosa, such as into bone, cartilage, or adjacent soft tissues.

### 2.3. Literature Search Strategy

Articles were searched systematically in Medline, Web of Science, and Embase until 10 January 2025. Mesh terms (‘Nasal Mucosa’, ‘Paranasal Sinuses’, ‘Nasal Cavity’), (‘Melanoma’, ‘Neoplasm Recurrence, Local’) and (‘Mortality’, ‘Prognosis’ and ‘Recurrence’) were included in the search strategy (Appendix A.1). No restriction was set on the language of the articles and translations were arranged when necessary. The search strategy was developed with the assistance of an information specialist from the library. Platforms & dates: MEDLINE (via PubMed), Web of Science (via Clarivate), and Embase (via Elsevier); last search on 10 January 2025; no language restrictions. Deduplication: Records were exported to EndNote 20 (Clarivate) and deduplicated using the Find Duplicates function with automated matching on Title, Authors, Year, and DOI; remaining duplicates were confirmed and removed by manual verification. Availability: Full, reproducible search strings for each database are provided in Appendix A.1.

### 2.4. Study Selection

Inclusion criteria: (1) the patients diagnosed with primary SNMM (2) the articles include tumor location or extension depiction (3) outcomes OS, progression-free survival (PFS), disease free survival (DFS), disease-specific survival (DSS), and disease control rate are included; (4) studies with original data, including prospective or retrospective cohort, random control study, case–control, comparative and case series (including at least 5 patients or more).

Exclusion criteria: (1) No description of tumor location or extension and outcomes; (2) Review articles (however, references were checked for original studies) or case reports (including fewer than 5 patients).

Additionally, the reference lists of included publications were checked for additional studies.

### 2.5. Data Extraction and Quality Assessment

Two reviewers (FY and MRC) independently screened all retrieved articles and assessed the methodological quality of the studies included in both the systematic review and meta-analysis. Disagreements were resolved through discussion, and if consensus could not be reached, a consensus meeting with a senior researcher (GBH) was held to resolve the discrepancy. Additionally, authors of the original publications were contacted for clarification and further data if the reported data were unclear.

For the systematic review, the extracted data included study characteristics such as the first author, country, year of publication, study design, treatment modalities, and sample size. Additionally, patient-related tumor and treatment characteristics, including age, gender, TNM stage, tumor site, treatment modalities, and outcome were also extracted. All recorded outcomes were systematically documented, including OS, DSS, and PFS, as reported in the studies included.

For the meta-analysis, a subset of studies was further analyzed quantitatively. Studies that might have overlap in the included cases were excluded and only one study with the largest case number was included to avoid duplicate cases in the meta-analysis. A modified version of the Quality in Prognosis Studies (QUIPS-2) [13,14,15] tool was used to evaluate the risk of bias in the included studies, recommended by the Cochrane Prognosis Methods Group. Two independent reviewers (FY and MRC) evaluated the risk of bias, resolving any discrepancies through consensus. If necessary, a third author (IW) facilitated the resolution process.

### 2.6. Statistical Analysis

In the systematic review, only descriptive statistics were used.

In the meta-analysis, the effects of different tumor locations/extensions on OS were analyzed, including nasal cavity, paranasal sinuses, maxillary sinus, ethmoid sinus, orbital involvement, and bone involvement. Hazard ratios (HRs) with 95% confidence intervals (CIs) were computed to evaluate OS. The heterogeneity among the studies was assessed via the *I*^2^ statistic, with *I*^2^ > 50% indicating substantial heterogeneity. For the χ^2^ test, a *p* < 0.10 was considered indicative of significant heterogeneity. If significant heterogeneity was found in the included studies, a random effect model was used to pool data. Otherwise, a fixed effect model was used.

All analyses were performed using Stata 12.0 software. A two-tailed statistical test was conducted, with *p* < 0.05 considered statistically significant. Subgroup analysis requires that the number of studies in each group be at least 2 [16]. Sensitivity analysis was conducted to explore potential sources of heterogeneity.

Small-study effects/publication bias were planned to be assessed using Begg’s and Egger’s tests when a synthesis included ≥10 studies; otherwise, these tests were not performed due to low power.

All analyses were conducted in Stata 12.0 (StataCorp). We did not perform a formal certainty-of-evidence (GRADE) assessment because all included evidence was observational with heterogeneous definitions of the prognostic factors; this is acknowledged as a limitation.

## 3. Results

### 3.1. Study Selection

After deduplication, 1314 records were screened at the title/abstract level. In addition, 1 record was identified through citation searching. In total, 224 reports were sought for retrieval (223 from databases plus 1 from citation searching); 2 could not be retrieved, leaving 222 reports for full-text assessment. Thirty-four studies met the eligibility criteria and were included in the systematic review, of which 10 contributed data to the meta-analysis. Reasons for excluding 188 reports at full-text assessment are detailed in Figure 1 (PRISMA 2020 flow diagram).

### 3.2. Study Characteristics

Table S1 summarizes the detailed study characteristics of all included articles in the systematic review. Studies, also included in the meta-analysis, are shown with a hash. In total, 48 studies were included. The sample size of individual studies ranged from 10 to 1874 patients. Most studies were retrospective and single-center. The analyzed tumor sites included the nasal cavity, maxillary sinus, ethmoid sinus, as well as cases with orbital or bone involvement. The proportion of female patients ranged from 29% to 71%, and the mean or median age of participants varied between 55 and 73 years. The follow-up duration ranged from 1 to 273 months across studies. OS was the most frequently reported outcome, followed by DSS, DFS and PFS.

### 3.3. Risk of Bias

Risk of bias was assessed using the QUIPS-2 tool across six domains (Table A1). Most studies showed low to moderate risk overall. Study participation was mainly low or moderate risk, with one high. Attrition varied, with several studies rated high risk. Prognostic factors and outcome measurements were generally low risk. Confounding was often moderate to high risk, indicating challenges in controlling biases. Statistical analysis and reporting were mostly low or moderate risk. These results suggest moderate overall study quality.

### 3.4. Systematic Review

Research consistently indicates that SNMM is more frequently located in the nasal cavity than in the paranasal sinuses [3,17,18,19]. However, while most findings are consistent, studies on tumor site/extension and survival have produced varying results. Furthermore, the description of the tumor site and extension is highly varied among studies. Evidence suggests that tumors arising in the paranasal sinuses, particularly the maxillary and ethmoid sinuses, are associated with worse survival outcomes compared to those confined to the nasal cavity [5,11,17,19,20,21,22,23,24,25,26,27,28,29,30]. However, some reports have not found any survival differences between SNMM originating from the nasal cavity and those from the paranasal sinuses [18,31,32,33,34].

Several studies have reported that SNMM involving the maxillary sinus exhibits poorer survival rates [6,10,35]. Similarly, ethmoid sinus involvement has been linked to decreased survival [10], likely due to its anatomical location and increased likelihood of local invasion. Low et al. found that the maxillary sinus, frontal sinus, and overlapping lesions of accessory sinuses were significantly associated with worse OS and DSS compared to the nasal cavity, whereas no significant difference in OS or DSS was observed for the ethmoid sinus [12].

Furthermore, tumor extension beyond the primary site significantly impacts prognosis [36,37]. Extension into the sphenoid sinus has been identified as a negative prognostic factor, correlating with lower survival rates [38]. Orbital and anterior cranial fossa involvement also appears to worsen outcomes [10], further highlighting the importance of tumor location and spread in SNMM prognosis.

### 3.5. Meta-Analysis Outcomes

#### 3.5.1. Paranasal Sinus vs. Nasal Cavity

- Overall Analysis

**Figure 2:**
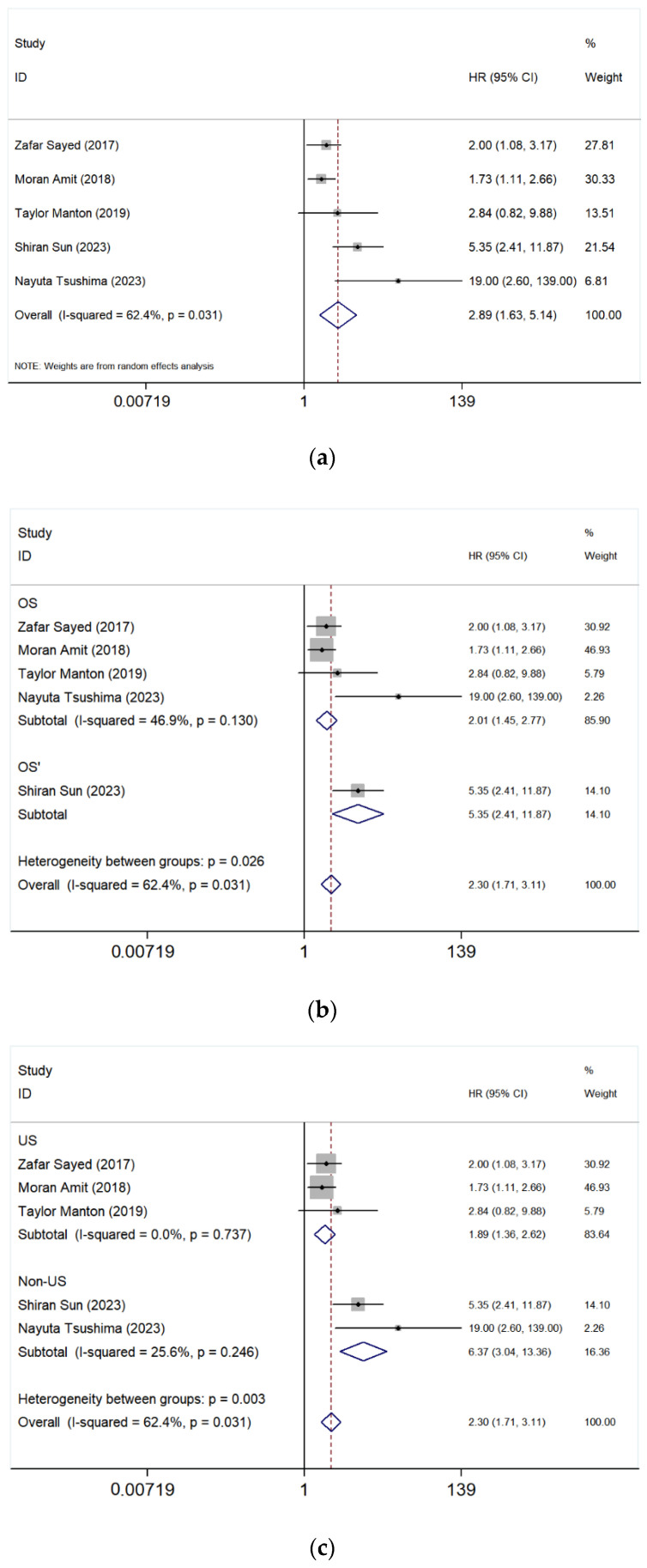
Forest plot comparing the risk of severe outcomes in patients with tumors originating in the paranasal sinus versus the nasal cavity. Studies included in the figure: Zafar Sayed et al. (2017) [20], Moran Amit et al. (2018) [21], Taylor Manton et al. (2019) [39], Shiran Sun et al. (2023) [17], Nayuta Tsushima et al. (2023) [4]. Grey squares represent individual study HRs; horizontal lines represent 95% CIs; blue diamond indicates pooled HRs; dashed vertical line indicates HR = 1. HR > 1 indicates poorer OS. (**a**) Patients with paranasal sinus involvement showed a 2.89-fold increased risk of poor prognosis (95% CI: 1.63–5.14). Due to high heterogeneity (*I*^2^ = 62.4%, *p* = 0.031), a random effect model was applied; (**b**) The same analysis after excluding one article in the sensitivity analysis; (**c**) A subgroup analysis was conducted based on geographic location (US vs. non-US).

The meta-analysis of five studies [4,17,20,21,39] comparing paranasal sinuses and nasal cavity involvement in SNMM showed a pooled HR of 2.89 (95% CI: 1.63–5.14, *p* = 0.031), indicating a significantly worse OS in patients with tumors located in the paranasal sinuses (Figure 2a). The heterogeneity was high (*I*^2^ = 62.4%), suggesting some variability among studies.

- Subgroup Analysis After Excluding the study of Sun et al. [17]

To assess the robustness of our findings, a sensitivity analysis was conducted by excluding the study by Sun et al. [17], which had both a high HR and weight. The revised pooled HR for OS was 2.01 (95% CI: 1.45–2.77, *I*^2^ = 46.9%, *p* = 0.130), suggesting that paranasal sinus involvement remained a significant predictor of worse survival outcomes (Figure 2b). The heterogeneity decreased compared to the overall analysis (*I*^2^ = 46.9% vs. 62.4%), indicating that the Sun et al. study contributed substantially to variability. These results suggest that while the effect remains significant, variations between studies impact the overall conclusions, highlighting the need for further research with standardized methodologies.

- Subgroup Analysis by Geographic Region

A geographic subgroup analysis was conducted to compare US-based and non-US-based studies. The pooled HR for US studies was 1.89 (95% CI: 1.36–2.62, *I*^2^ = 0.0%, *p* = 0.737), while for non-US studies, it was significantly higher at 6.37 (95% CI: 3.04–13.36, *I*^2^ = 25.6%, *p* = 0.246) (Figure 2c). These findings suggest potential regional differences in prognosis, possibly due to variations in treatment approaches, healthcare systems, or patient characteristics.

#### 3.5.2. Maxillary Sinus vs. Nasal Cavity

**Figure 3:**
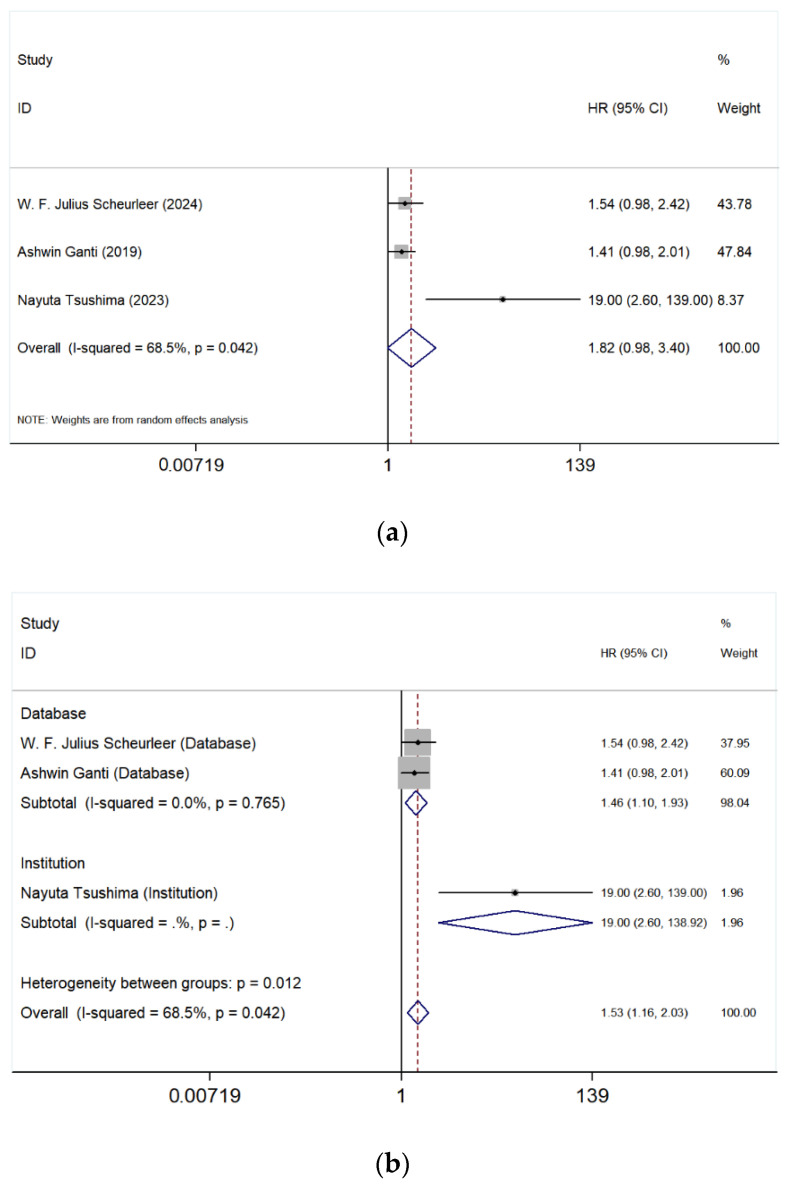
Forest plot analyzing the impact of tumor location in the maxillary sinus compared to the nasal cavity. Studies included in the figure: W. F. Julius Scheureler et al. (2024) [6], Ashwin Ganti et al. (2019) [3], Nayuta Tsushima et al. (2023) [4]. Grey squares represent individual study HRs; horizontal lines represent 95% CIs; blue diamond indicates pooled HRs; dashed vertical line indicates HR = 1. HR > 1 indicates poorer OS. (**a**) The risk of adverse outcome was 1.82-fold higher in patients with maxillary sinus involvement (95% CI: 0.98–3.40). Substantial heterogeneity was present (*I*^2^ = 68.5%, *p* = 0.042), thus the random effect model was used; (**b**) A subgroup analysis was conducted based on data source (database vs. institutional).

The meta-analysis of three studies [3,4,6] compared the prognostic differences between the maxillary sinus and the nasal cavity. The first figure (Figure 3a) shows a pooled HR of 1.82 (95% CI: 0.98–3.40) with an *I*^2^ value of 68.5% (*p* = 0.042), indicating high heterogeneity among the studies. Although the pooled HR suggests a trend toward worse OS for tumors originating in the maxillary sinus compared to the nasal cavity, the confidence interval crosses 1, indicating that the difference is not statistically significant.

Further stratified analyses based on data source (Database) and institutional studies (Institution) were performed (Figure 3b). In the database group [3,6], the pooled HR was 1.46 (95% CI: 1.10–1.93), with an *I*^2^ value of <0.0% (*p* = 0.765), suggesting no significant heterogeneity within this group. However, the HR for the institutional study [4] analyzed separately was 19.00 (95% CI: 2.60–138.92). This analysis suggests that findings from database studies were more consistent, whereas the HR value from the institutional study was significantly higher than that of the database studies, which may be the primary contributor to the overall high heterogeneity.

#### 3.5.3. Ethmoid Sinus vs. Nasal Cavity

**Figure 4:**
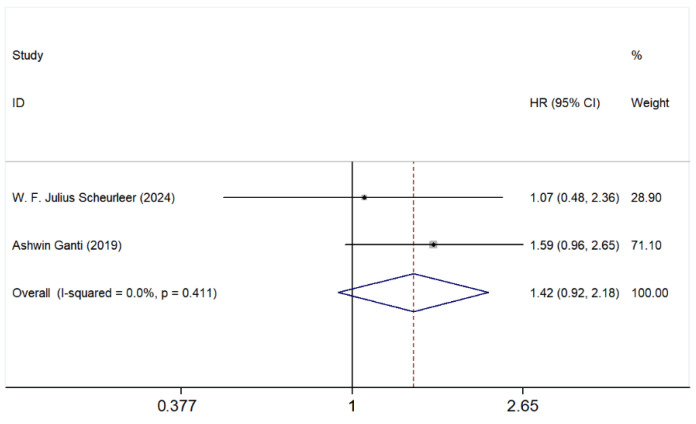
Risk comparison of patients with tumors in the ethmoid sinus versus the nasal cavity. Studies included in the figure: W. F. Julius Scheureler et al. (2024) [6], Ashwin Ganti et al. (2019) [3]. Grey squares represent individual study HRs; horizontal lines represent 95% CIs; blue diamond indicates pooled HRs; dashed vertical line indicates HR = 1. HR > 1 indicates poorer OS. The pooled HR indicated a 1.42-fold elevated risk in the ethmoid group (95% CI: 0.92–2.18), with low heterogeneity across studies (*I*^2^ = 0.0%, *p* = 0.411), supporting the use of a fixed effect model.

As shown in Figure 4, the pooled HR was 1.42 (95% CI: 0.92–2.18), suggesting a potential but non-significant increase in risk for patients with ethmoid sinus involvement compared to the nasal cavity. The heterogeneity across studies was low, with an *I^2^* value of 0.0% (*p* = 0.411), indicating consistency among the included studies.

Individually, Scheurleer et al. [6] reported an HR of 1.07 (95% CI: 0.48–2.36), while Ganti et al. [3] showed an HR of 1.59 (95% CI: 0.96–2.65). The larger weight of Ganti’s study (71.10%) suggests that it had a greater influence on the pooled estimate.

#### 3.5.4. Orbital Involvement

**Figure 5:**
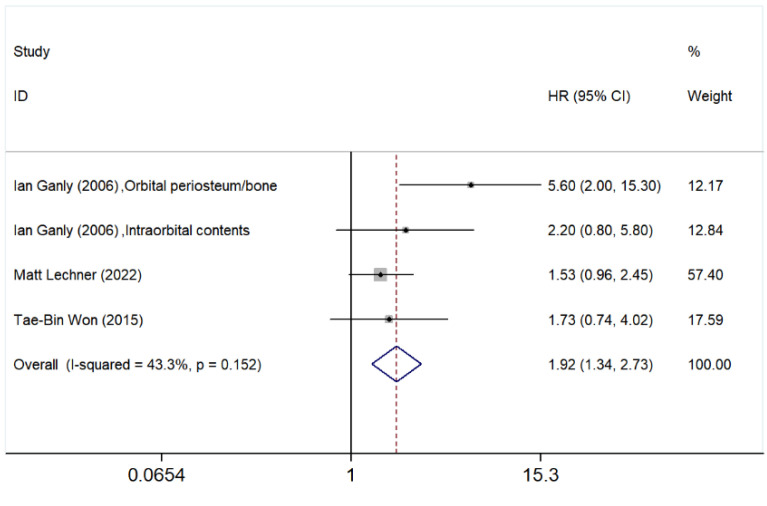
Forest plot demonstrating the risk of poor outcomes in patients with orbital involvement compared to those without. Studies included in the figure: Ian Ganly et al. (2006) [40]; Matt Lechner et al. (2022) [41]; Tae-Bin Won et al. (2015) [22]. Grey squares represent individual study HRs; horizontal lines represent 95% CIs; blue diamond indicates pooled HRs; dashed vertical line indicates HR = 1. HR > 1 indicates poorer OS. Orbital extension was associated with a significantly higher risk (HR = 1.92, 95% CI: 1.34–2.73). A fixed effect model was employed due to moderate heterogeneity (*I*^2^ = 43.3%, *p* = 0.152).

In addition to the paranasal sinus, the effect of orbital involvement on SNMM patients’ OS was also explored. The meta-analysis presented in Figure 5 evaluates the impact of orbital involvement on patient outcomes. The pooled HR for OS was 1.92 (95% CI: 1.34–2.73), suggesting that orbital involvement is associated with a significantly increased risk of poor prognosis.

Among individual studies, Ganly et al. [40] reported HRs of 5.60 (95% CI: 2.00–15.30) for orbital periosteum/bone involvement and 2.20 (95% CI: 0.80–5.80) for intraorbital content invasion. Meanwhile, Lechner et al. [41] and Won et al. [22] reported HRs of 1.53 (95% CI: 0.96–2.45) and 1.73 (95% CI: 0.74–4.02), respectively. These findings indicate variability in the impact of different degrees of orbital invasion on survival.

Although the heterogeneity analysis (*I*^2^ = 43.3%, *p* = 0.152) suggests moderate variability among the included studies, the *p*-value did not meet the pre-specified threshold (*p* < 0.10). Therefore, a fixed effect model was applied in accordance with the study protocol.

#### 3.5.5. Bone Involvement

**Figure 6:**
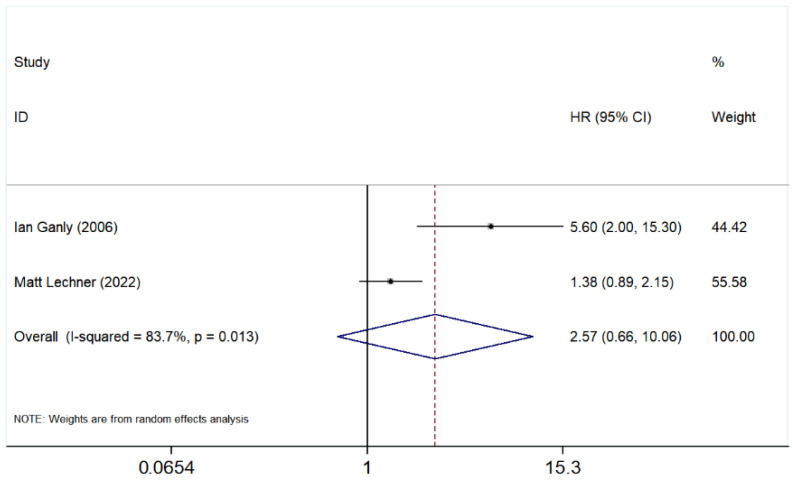
Forest plot evaluating the prognostic impact of bone invasion. Studies included in the figure: Ian Ganly et al. (2006) [40]; Matt Lechner et al. (2022) [41]. Grey squares represent individual study HRs; horizontal lines represent 95% CIs; blue diamond indicates pooled HRs; dashed vertical line indicates HR = 1. HR > 1 indicates poorer OS. The association between bone involvement and OS was not statistically significant (HR = 2.57, 95% CI: 0.66–10.06). Given the heterogeneity (*I*^2^ = 83.7%, *p* = 0.013), a random effect model was applied.

The pooled HR for OS was 2.57 (95% CI: 0.66–10.06), indicating a potential association between bone invasion and poorer prognosis, though the confidence interval suggests high uncertainty in the estimate (Figure 6).

Individually, Ganly et al. [40] reported an HR of 5.60 (95% CI: 2.00–15.30), suggesting a significant negative impact of bone involvement on survival. In contrast, Lechner et al. [41] reported a lower HR of 1.38 (95% CI: 0.89–2.15), indicating a more moderate effect.

The heterogeneity analysis showed a high *I*^2^ value of 83.7% (*p* = 0.013), suggesting substantial variability between the studies. A random effects model was applied to account for this heterogeneity. Overall, while bone involvement appears to be associated with worse survival outcomes, the wide confidence interval and high heterogeneity highlight the need for further studies to confirm these findings.

## 4. Discussion

Research shows that SNMM is more commonly located in the nasal cavity than in the paranasal sinuses [3,18]. However, studies on tumor sites and survival outcomes are inconsistent, with some indicating worse survival for paranasal sinus tumors, while others show no significant survival differences [31,32]. Similarly, studies examining the extension of tumors to adjacent anatomical structures and their impact on survival have also reported conflicting results [22,40,41]. Several factors may be attributed to this discrepancy, reflecting the underlying heterogeneity among the included studies. High *I*^2^ values observed in several comparisons likely reflect heterogeneity in patient selection, case definitions, sample size, and treatment strategies across studies. Variations in the adoption of ICIs (introduced after 2014) may also contribute to outcome differences. First, variations in sample size might have led to insufficient statistical power to detect survival differences [18,31,32,33,34]. Second, variations in treatment strategies across studies, such as the extent of surgical resection and the application of adjuvant treatment, may influence survival outcomes independently of tumor location. Moreover, differences in follow-up duration and data incompleteness across studies may impact survival analyses, particularly in retrospective cohorts.

As the first meta-analysis focusing on the association between SNMM tumor location/extension and survival, it evaluates the prognostic significance of different anatomical sites of tumor involvement, including primary tumor site, such as paranasal sinuses (Figure 2), maxillary sinus (Figure 3), ethmoid sinus (Figure 4), and tumor extension such as orbital involvement (Figure 5), and bone invasion (Figure 6). We found that both paranasal sinus and orbital involvement were identified as significant and clinically relevant adverse prognostic factors for OS in SNMM patients. However, maxillary sinus and ethmoid sinus involvement individually, and bone invasion were not statistically significant, but there is a noticeable trend suggesting their potential negative impact. Unfortunately, no further firm conclusions can be drawn or prediction models can be developed based on the published literature data, as the way of reporting the findings in individual studies is highly diverse.

We found that patients with tumors located in paranasal sinuses had a worse prognosis compared to those with tumors in the nasal cavity, and this is consistent with the previous reports [5,11,12,23,42]. However, it is somewhat confusing that the results for the maxillary sinus and ethmoid sinus were not statistically significant despite the overall significance observed for the paranasal sinuses. This discrepancy may be attributed to the limited number of included studies. Nonetheless, as the HRs are greater than 1, these tumor locations may still be considered negative prognostic factors for SNMM patients.

As reported previously, SNMM originating from the maxillary sinus [12,35,42] or ethmoid sinus [42] is associated with a worse prognosis. In addition, Koivunen et al. reported that tumor extension into the sphenoid sinus significantly reduces survival [38], and involvement of the frontal sinus is also associated with poorer survival outcomes [12,38].

The adverse prognosis associated with paranasal sinus involvement may be explained by the proximity of the sinuses to critical anatomical structures, such as the orbit, skull base, and internal carotid artery [43], combined with their complex anatomy, which makes achieving oncologic resection with clear margins particularly challenging. Moreover, tumors originating in the paranasal sinuses may be diagnosed at a more advanced stage compared to those in the nasal cavity, as they tend to cause symptoms such as nasal obstruction or epistaxis later in the course of the disease. This delay in clinical detection may contribute to their poorer prognosis.

These findings underscore the negative prognostic impact of paranasal sinus involvement in SNMM, highlighting the need for validation in larger cohorts. Although results for the maxillary and ethmoid sinuses were not statistically significant, methodological differences and population heterogeneity may have contributed. The observed HRs suggest a trend toward worse prognosis, particularly for the ethmoid sinus, warranting further investigation to refine risk stratification in SNMM patients.

Due to the highly aggressive nature of SNMM, some patients, especially those with advanced-stage disease, exhibit orbital invasion [44,45,46,47]. For patients with tumor involvement of the orbit, surgical resection is highly mutilating. According to our analysis, orbital involvement is significantly associated with a higher risk of poor prognosis. Ganly et al. reported that the extent of orbital involvement was an independent predictor of both DSS and OS [40]. Surprisingly, this study showed that patients with orbital content involvement had better prognoses than those with only periosteal/bone invasion [40]. The authors attributed this controversial finding to a treatment bias: patients with orbital content involvement were more frequently managed by surgical orbital exenteration, whereas eye-sparing approaches were preferred for those with only periosteal or bony invasion. This is a very good example of bias, which may be true for all retrospective studies. Additionally, recent research suggests the potential existence of previously unrecognized lymphatic drainage pathways in the orbital region, through which SNMM may undergo lymphogenic metastasis via the retrobulbar fat [46].

Prasad et al. proposed a three-level microstaging system for HNMM patients based on prognosis. They classified invasion into bone, cartilage, or deep skeletal muscle as the highest level (level III), which indicates a poorer prognosis [48]. Although the meta-analysis did not identify bone involvement as a significant predictor of poorer prognosis, this is likely due to the limited number of included studies. In the study by Manton et al. involving 31 patients, they found that bone invasion had no significant impact on OS or locoregional control in SNMM patients but was significantly associated with an increased risk of distant metastasis, which in turn could contribute to worse survival outcomes [39]. Ganly et al. found that bone invasion was associated with worse prognosis than orbital involvement in 53 patients undergoing craniofacial resection [40]. This may be because orbital exenteration is performed for intraorbital invasion, while orbital preservation is attempted for bone involvement. Patients with periosteum/bone involvement who did not undergo orbital exenteration may experience higher recurrence rates and poorer outcomes [40].

In the current Union for International Cancer Control (UICC) classification of malignant melanoma of the upper aerodigestive tract, including SNMM, as well as other staging systems, the criteria for evaluating disease extent do not include tumor involvement in adjacent anatomical sites, such as paranasal sinuses and orbital involvement. However, our meta-analysis results indicate that anatomical location is a significant factor influencing the OS of SNMM patients. So far, there have been previous studies that supported a stratified staging system incorporating, among others, anatomical involvement [11,41,49,50]. Therefore, based on our findings and in line with previous research, including the anatomical location of and/or extension to the paranasal sinuses and to the orbit, may be considered to include in the staging system to achieve a more informative risk stratification. Our findings suggest that anatomical factors such as paranasal sinus and orbital involvement should be explicitly incorporated into future AJCC/UICC staging revisions for SNMM, as they provide meaningful prognostic discrimination. Clinically, identifying these features preoperatively could guide surgical planning and the use of adjuvant therapies. Moreover, better estimation of prognosis may also help in shared decision-making.

In this meta-analysis, several limitations should be acknowledged.

First, the number of studies included in each subgroup analysis was relatively small, limiting the statistical power. Given fewer than 10 studies per outcome, tests for small-study effects/publication bias (Begg’s/Egger’s) and funnel plots were underpowered and therefore were not performed. To address this limitation, we carefully examined study characteristics, including sample size, outcomes, and treatment modalities mentioned in the methodological part, and completeness of reporting. Despite these efforts, the possibility of publication bias cannot be entirely ruled out. Although small-study effect tests were underpowered due to limited study numbers, this limitation may reduce the sensitivity to detect publication bias, warranting cautious interpretation of pooled estimates.

Second, the retrospective nature of the included studies may introduce selection bias, leading to heterogeneity in patient populations and treatment protocols. This variability could compromise the reliability of survival estimates and obscure the true prognostic value of tumor location and extension.

Another limitation of this meta-analysis is that the potential co-dependency between bone and orbital invasion was not investigated, as these features were analyzed independently in the included studies. Future analyses accounting for potential interrelationships between these factors could provide a more comprehensive understanding of their prognostic significance.

Another important limitation is the heterogeneity among the included studies regarding staging systems, surgical management, margin status, and the use of adjuvant or systemic therapies. These factors are known to independently influence survival in SNMM and may therefore act as confounding variables in our analysis. Most included studies did not uniformly report TNM staging or provide individual patient-level data that would allow pooled multivariate analysis. Because of this incomplete and inconsistent reporting, subgroup or meta-regression analyses were not feasible. Future prospective multicenter studies collecting standardized clinicopathologic and molecular data are essential to confirm the independent prognostic value of tumor location and extension. Such efforts would enable more robust prognostic modeling and could help to integrate anatomical and clinical variables into an improved staging framework for SNMM.

Additionally, distinguishing the exact site of tumor origin in SNMM is often challenging for clinicians due to the presentation typically in advanced stages when the tumor has already spread over several (sub)sites. These tumors are typically asymptomatic in their early stages, causing patient’s delay. Moreover, due to atypical primary symptoms, there is often a significant diagnostic (doctor’s) delay. This can lead to inconsistencies in tumor classification across studies and contribute to the heterogeneity observed in the meta-analysis. Future research should focus on refining classification criteria and incorporating advanced imaging or molecular profiling techniques to improve diagnostic accuracy and better guide treatment strategies.

Finally, we did not undertake a formal GRADE assessment because all included evidence was observational, and definitions of the prognostic factors varied across studies; we acknowledge this as a limitation.

## 5. Conclusions

This meta-analysis found that paranasal sinuses and orbital involvement are significant negative prognostic factors for SNMM. Involvement of the maxillary sinus, ethmoid sinus, and bone tends to be associated with poorer outcomes in SNMM; however, the limited number of included studies restricts the robustness of these findings, requiring further validation with larger cohorts.

The results of this study may contribute to a more accurate staging system, which may lead to better risk stratification and improved treatment strategies for SNMM patients. Besides clinical parameters, tumor characteristics should also be included in future prognostic models, similar to how HPV status is incorporated into the stratification of oropharyngeal cancer. Our future work will also focus on exploring molecular biomarkers of SNMM. Discovering molecular mechanisms, driving SNMM will provide insights into personalized treatment strategies for patients with this rare and aggressive cancer.

## Data Availability

All data analyzed in this study were obtained from previously published studies, which are cited in the article. No new data was generated in this study.

## Supplementary Materials

The following supporting information can be downloaded at: https://www.mdpi.com/article/10.3390/cancers17233757/s1, Table S1: Characteristics and results of studies included in systematic review and meta-analysis (#).

## Abbreviations

The following abbreviations are used in this manuscript:

SNMM	Sinonasal mucosal melanoma
HR	Hazard ratio
CI	Confidence interval
OS	Overall survival
S	Surgery
CFR	Craniofacial resection
Gy	Gray
PORT	Postoperative radiotherapy
ICI	Immune checkpoint inhibitor
TNM	Tumor Node Metastasis
PFS	Progression-free survival
DFS	Disease-free survival
DSS	Disease-specific survival
MM	Mucosal melanoma
HNMM	Head and neck mucosal melanoma
MSS	Melanoma-specific survival
RFS	Recurrence-free survival
LR	Local recurrence
RR	Regional recurrence
DM	Distant metastasis
RS	Relative survival
POCRT	Postoperative chemoradiotherapy
RT	Radiotherapy
SD	Standard deviation
IQR	Interquartile range
NR	Not reported
NCDB	National Cancer Database
SEER	Surveillance, Epidemiology, and End Results database
NCR	Netherlands Cancer Registry
REFCOR	Réseau d’Expertise Français sur les Cancers ORL Rares
NOS	Not otherwise specified

## Appendix A

### Appendix A.1. Search Strategy

The systematic literature search was conducted in Medline (via PubMed), Web of Science, and Embase databases. The full search strategies are detailed below. These searches were performed to identify studies on sinonasal mucosal melanoma and its prognostic outcomes.

Medline:

(“Nasal Mucosa”[Mesh] OR “Paranasal Sinuses”[Mesh] OR “Nasal Cavity”[Mesh] OR “nasal cavit*”[tiab] OR Paranasal*[tiab] OR ethmoid[tiab] OR “frontal sinus*”[tiab] OR “maxillary sinus*”[tiab] OR sphenoid*[tiab])

AND

(“Melanoma”[Mesh] OR melanoma*[tiab] OR “lentigo maligna*”[tiab] OR “Malignant Lentigo*”[tiab])

AND

(“Mortality”[Mesh] OR “Prognosis”[Mesh] OR “Recurrence”[Mesh] OR “Neoplasm Recurrence, Local”[Mesh] OR Prognos*[tiab] OR mortal*[tiab] OR surviv*[tiab] OR recurren*[tiab] OR progress*[tiab] OR metastas*[tiab])

Web of Science:

TS = (“Nasal Mucosa” OR “Paranasal Sinuses” OR “Nasal Cavity” OR “nasal cavit*” OR Paranasal* OR ethmoid OR “frontal sinus*” OR “maxillary sinus*” OR sphenoid*) AND TS = (“Melanoma” OR melanoma* OR “lentigo maligna*” OR “Malignant Lentigo*”) AND TS = (“Mortality” OR “Prognosis” OR “Recurrence” OR “Neoplasm Recurrence, Local” OR Prognos* OR mortal* OR surviv* OR recurren* OR progress* OR metastas*)

Embase:

(‘nasal mucosa’/exp OR ‘paranasal sinus’/exp OR ‘nasal cavity’/exp OR ‘nasal cavit*’:ti,ab OR ‘paranasal*’:ti,ab OR ‘ethmoid’:ti,ab OR ‘frontal sinus*’:ti,ab OR ‘maxillary sinus*’:ti,ab OR ‘sphenoid*’:ti,ab)

AND

(‘melanoma’/exp OR melanoma*:ti,ab OR ‘lentigo maligna*’:ti,ab OR ‘malignant lentigo*’:ti,ab)

AND

(‘mortality’/exp OR ‘prognosis’/exp OR ‘recurrence’/exp OR ‘neoplasm recurrence, local’/exp OR prognos*:ti,ab OR mortal*:ti,ab OR surviv*:ti,ab OR recurren*:ti,ab OR progress*:ti,ab OR metastas*:ti,ab)

### Appendix A.2. Risk of Bias Assessment

Risk of bias was assessed using the QUIPS-2 tool, which evaluates study quality in six domains: Study Participation, Study Attrition, Prognostic Factor Measurement, Outcome Measurement, Study Confounding, and Statistical Analysis and Reporting. The assessment for each included study is summarized in Table A1.

**Table A1:** QUIPS-2 risk of bias assessment for included studies.

Study	Study Participation	Study Attrition	Prognostic Factor Measurement	Outcome Measurement	Study Confounding	Statistical Analysis and Reporting
Sayed et al., 2017. [20]	Moderate	Low	Low	Low	Moderate	Low
Ganly et al., 2006. [40]	Moderate	Low	Moderate	Low	Moderate	Low
Lechner et al., 2022. [41]	Low	Moderate	Low	Low	Moderate	Low
Amit et al., 2018. [21]	Low	Low	Low	Low	Low	Low
Manton et al., 2019. [39]	Low	High	Low	Low	Moderate	Low
Scheurleer et al., 2024. [6]	Low	High	Low	Low	High	Moderate
Ganti et al., 2020. [3]	Low	High	Low	Low	Moderate	Low
Won et al., 2015. [22]	Low	High	Low	Low	Moderate	Moderate
Sun et al., 2023. [17]	Low	High	Low	Low	Low	Low
Tsushima et al., 2023. [4]	Low	High	Low	Low	Moderate	Low
Koivunen et al., 2012. [38]	Low	Low	Moderate	Low	High	Moderate
Yang et al., 2022. [11]	Low	Low	Moderate	Low	Low	Low
Gras-Cabrerizo et al., 2015. [31]	Moderate	Low	Low	Low	Moderate	Low
Abt et al., 2021. [23]	Low	Moderate	Low	Low	Moderate	Low
Low et al., 2020. [12]	Low	Low	Moderate	Low	Moderate	Low
Moya-Plana et al., 2019. [24]	Low	Low	Low	Low	Low	Low
Roth et al., 2010. [10]	Moderate	Low	Moderate	Low	High	Moderate
Martin et al., 2004. [32]	Moderate	Low	Low	Low	Moderate	Low
Wang et al., 2022. [18]	Low	Moderate	Low	Low	Moderate	Low
Houette et al., 2016. [25]	Moderate	Low	Low	Low	High	Moderate
Lundberg et al., 2019. [26]	Low	Low	Low	Low	Moderate	Moderate
Letievant et al., 2016. [19]	Moderate	Moderate	Moderate	Low	High	Moderate
Khan et al., 2014. [42]	Low	Low	Low	Low	Moderate	Low
Wang et al., 2020. [27]	Low	Moderate	Low	Low	Moderate	Moderate
Dréno et al., 2017. [28]	Moderate	Moderate	Low	Low	High	Moderate
Vandenhende et al., 2012. [36]	Moderate	Moderate	Low	Low	High	Moderate
Liétin et al., 2010. [37]	High	Moderate	Low	Low	High	Moderate
Rojas-Lechuga et al., 2022. [33]	Low	Low	Low	Low	Low	Low
Amit et al., 2017. [5]	Low	Low	Low	Low	Moderate	Low
Sun et al., 2014. [34]	Low	Low	Low	Low	Moderate	Low
Dauer et al., 2008. [35]	Moderate	High	Moderate	Low	Moderate	Moderate
Ajmani et al., 2017. [29]	Low	Low	Low	Low	Moderate	Low
Zhu et al., 2024. [49]	Low	Low	Low	Low	Low	Low
Yu et al., 2015. [30]	Moderate	Low	Moderate	Low	High	Moderate
