# Supplementary material for: The Effect of Tumor Location and Extension on Survival in Patients with Sinonasal Mucosal Melanoma: A Systematic Review and Meta-Analysis
Source: Cancers (Basel). 2025 Nov 25;17(23):3757. doi: 10.3390/cancers17233757 (PMC12691534; doi:10.3390/cancers17233757)
Supplement: Supplementary file 1 [file cancers-17-03757-s001.zip › cancers-3966393-supplementary.pdf]

Table S1. Characteristics and results of studies included in systematic review and meta-analysis (#).

Author	Country	Source of patients	Diagnosed/Treatment period	Follow-up (months)	Sample size	Study design	Inclusion criteria	Age(years)	Female (%)	Primary site/extension	Outcome	Treatment
#Sayed et al, 2017. [17]	US	Single center	1997-2015	NR	72	Retrospective cohort study	SNMM, nasal cavity, paranasal sinuses, N0M0	mean 67 (SD 12)	54	Primary site: 50 Nasal cavity, 22 Sinus.	3-year OS: 52%.	52 open/CFR S, 20 endoscopic S
#Ganly et al, 2006. [18]	US	Multicenter: International	NR	median 10 (range 1-159)	53	Retrospective cohort study	Craniofacial resection for MM of the skull base	median 63 (range 3-81)	30	53 Anterior cranial fossa invasion, 32 Orbital involvement, 19 Intracranial involvement	3-year OS: 28.2%, 3-year DSS: 29.7%, 3-year RFS: 25.5%.	53 CRF, 22 adjuvant RT, 3 adjuvant chemotherapy
#Lechner et al, 2022. [19]	UK&US	Multicenter: International	1999-2021	median 21.3	505	Retrospective cohort study	SNMM	median 67 (range 15-93)	53.7	Site involvement of original tumor at presentation: 411 Nasal involvement, 199 Sinus involvement, 65 Skull base involvement, 15 Intracranial involvement.	1-year OS 77.6%, 3-year OS 49.2%, 5-year OS 38.3%. 1-year DFS 61.4%, 3-year DFS 30.6%, and 5-year DFS 22.0%.	197 S only, 215 S + adjuvant RT, 54 adjuvant chemotherapy
#Amit et al, 2018. [20]	US	Single center	1991.1.1-2016.12.31	mean 47 (SD 4, range 1.1-220)	198	Retrospective cohort study	SNMM	mean 65 (SD 11, range 34-94)	43	Primary site: 142 Nasal cavity, 56 Paranasal sinus.	5-year OS: 38%, 5-year DSS: 58%, 5-year DFS 27%.	170 S, 71 PORT, 34 POCRT, 14 systemic treatment, 14 chemoradiation therapy
#Manton et al, 2019. [21]	US	Single center	2009-2017	median 38.5	31	Retrospective cohort study	SNMM	mean71 (range 52-85)	71	Primary site: 23 Nasal cavity, 8 Paranasal sinuses	2-year OS: 77%.	All S (26 adjuvant RT, 3 immunotherapy for distant metastatic disease)
#Scheurleer et al, 2024. [6]	Netherlands	Database: NCR database	2001.1.1-2021.12.31	median (IQR): 20.2 (8.3-43.5)	320	Retrospective cohort study using a population-based database	SNMM	median (IQR): 73 (65-81.5)	55	Primary site: 262 Nasal cavity, 32 Maxillary sinus, 14 Ethmoid sinus, 3 Sphenoid sinus, 2 Frontal sinus, 7 Sinonasal NOS	5-year OS: 24.5%, 5-year RS: 32.4%.	63 S, 176 S + RT, 6 S + systemic (+RT), 32 RT, 9 systemic (+RT), 34 none/best supportive care
#Ganti et al, 2020. [3]	US	Database: NCDB	2004.1-2015.12	NR	1874	Retrospective cohort study using a population-based database	SNMM, nasal cavity, paranasal sinuses	mean 71	53	Primary site: 1312 Nasal cavity, 252 Maxillary sinus, 114 Ethmoid sinus, 7 Frontal sinus, 43 Sphenoid sinus, 146 Accessory sinuses	5-year OS: 24%.	S: 307 endoscopic, 390 open, 697 unknown; RT: 1093 RT, 715 no RT, 66 unknown; chemotherapy: 223

												chemotherapy, 1580 no chemotherapy
#Won et al, 2015. [22]	Korea	Multicenter: Korean	1994.4-2013.6	≥1 year; mean 40.9 (SD 38.95, range 12-200) (n=133, curative surgery).	155	Retrospective cohort study	Newly diagnosed SNMM patients with ≥1-year follow-up	mean 63.3 (range, 28-92)	48	Involved sites(n=133): 34 Maxillary sinus, 99 Nasal cavity (septum excluded), 54 Septum, 28 Ethmoid sinus, 6 Frontal sinus, 6 Sphenoid sinus, 6 Skull base, 9 Orbit, 5 Nasopharynx, 3 Nasolacrimal duct, 6 Skin.	3-year OS: 48.8%, 5-year OS: 40.1%.	Among 133 patients: all S, 54 PORT, 28 POST, 11 postoperative concurrent systemic therapy and RT
#Sun et al, 2023. [23]	China	Single center	1990.2-2020.8	median 118 (range 3-273)	107	Retrospective cohort study	SNMM	median (IQR): 58 (49-64)	49	Primary site: 96 Nasal cavity, 11 Paranasal sinus.	5-year OS, LR, RR, and DM rates were 40.1%, 43.9%, 20.1%, and 57.4%.	40 S, 63 S + RT, 4 RT
#Tsushima et al, 2023. [4]	Japan	Single center	2002.1-2021.4	median 2.2 years (range 0.5-8.6 years)	30	Retrospective cohort study	patients with previously untreated SNMM	median 72 (range 45-83)	40	28 Nasal cavity, 2 Paranasal sinus.	2-year OS: 70%, 5-year OS: 46%.	28 S, 28 PORT, 2 definitive RT
Koivunen et al, 2012. [24]	Finland	Multicenter: Finland	1990.1.1-2004.12.31	minimum 24, mean 37.1, median 30	50	Retrospective cohort study	SNMM	mean 70 (SD 10.9, range 46-93)	54	Primary site: 14 Nasal cavity (not septum), 6 Septum, 6 Ethmoid sinus, 6 Maxillary sinus, 18 Unknown. Involvement in Adjacent Anatomic Sites Not Considered in UICC: 11 Infraorbital,11 Sphenoid sinus, 4 Frontal sinus,6 Nasopharynx,5 Palate.	3-year OS: 44%, 5-year OS: 27%.	Treatment with curative intent was offered to 42 patients. 40 S, 7 RT only, 7 PORT
Yang et al, 2022. [11]	China	Database: SEER	1975-2015.12.31	minimum 6/ median of 15 (mean 21.7 ± 19.7, range 1-83)	305	Retrospective cohort study	SNMM, Nasal cavity, Paranasal sinuses	(training cohort, validation cohort): <50 (4.9%, 4.0%) / 50-70 (37.3%, 28.7%) / >70 (57.8%, 67.3%)	Training cohort: 50.5%; Validation cohort: 52.5%.	Primary site: 14 Ethmoid sinus, 53 Maxillary sinus, 238 Nasal cavity.	1-year OS: 66.2%, 2-year OS: 52.0%, 5-year OS: 25.5%.	229 RT, 251 S, 29 chemotherapy
Gras-Cabrerizo et al, 2015. [25]	Spain	Single center	1988 to 2013	minimum 12	20	Retrospective cohort study	SNMM	median 71 (range 42-87)	50	Primary site: 9 Ethmoidal sinus complex, 4 Inferior turbinate, 3 Nasal septum, 2	3-year OS: 47%, 5-year OS: 34%.	15 S + RT, 3 chemoradiotherapy, 2 S alone

									Maxillary sinus, 2 Nasal vestibule.		
Abt et al, 2021. [26]	US	Database: SEER	1973-2013	mean 2.6 years (SD 2.7)	928	Retrospective cohort study	SNMM, Nasal cavity, Paranasal sinuses	mean 70.6 (SD 13.7)	55	Primary site: 632 Nasal cavity, 302 Paranasal sinus.	5-year OS: 24.1%.	70 no treatment, 85 RT only, 299 S only, 437 S + RT
Low et al, 2020. [12]	US	Database: SEER	1973-2015	NR	382	Retrospective cohort study	SNMM, Nasal cavity, Paranasal sinuses	mean 69,4 (SD 13.4)	56	Primary site: 279 Nasal cavity, 60 Maxillary sinus, 18 Ethmoid sinus, 13 Sphenoid sinus, 11 Overlapping lesion of accessory sinuses, 1 Frontal sinus.	5-year OS: 22.7%, 5-year DSS: 26.8%.	NR
Moya-Plana et al, 2019. [27]	France	Database: REFCOR	2000-2017	median 45.5	226	Prospective cohort study	HNMM	mean 67.2 (range 28-96)	60	Primary site: 181 Nasal fossa, 45 Sinus.	In surgery group, 5-year OS:49.4%, 5-year PFS: 24.7%; no long-term survivors were observed when surgery was not feasible.	59 S only, 121 S + RT, 14 RT only, 31 others
Roth et al, 2010. [10]	Switzerland	Single center	1992-2007	at least 6	25	Retrospective cohort study	SNMM	mean 71 (range 40-94)	68	Primary site: 5 Ethmoid sinus, 5 Maxillary sinus, 7 Nasal cavity, 4 Septum, 4 Turbinate.	Median DFS 18 months, median OS 23 months. Median DFS: Ethmoid sinus 6 months, Maxillary sinus 2 months, Nasal cavity 25.5 months, Septum 17 months, Turbinate 16.5 months.	19 primary S with curative intention
Martin et al, 2004. [28]	Australia	Single center	1991-2002	median 79 (range 7-128)	20	Retrospective cohort study	SNMM	median 77 (range 45-91)	60	Site of involvement: 8 Nasal cavity, 9 Nasal cavity and paranasal sinus, 3 Paranasal sinus. Invasion: 10 None, 3 Soft tissue, 3 Soft tissue and orbital/intracranial, 2 Orbital/intracranial, 2 Unknown.	Median OS: 17 months, 2-year OS: 23%.	15 initial S followed by RT, 2 RT only, 2 radical S only, 1 S + adjuvant chemotherapy
Wang et al, 2022. [29]	China	Multicenter: China	2007.6-2018.6	median 21 (range 0-77)	117	Retrospective cohort study	SNMM	median 60 (SD 10, range 37-83)	51	Primary site: 84 Nasal cavity, 16 Nasal cavity and maxillary sinus, 7 Nasal cavity and ethmoid sinus, 10 Others.	Median survival time in 106 patients: 21 months (range 0-77	NR

										months). The 1-, 2-, 3- and 5-year survival rates were 78.6%, 36.8%, 17.1% and 6.8%.	
Houette et al, 2016. [30]	France	Single center	1998.8-2014.6	mean 40	18	Retrospective cohort study	SNMM	mean 72 (range 54-94)	61	Primary site: 7 Nasal septum, 5 Inferior turbinate, 5 Sinus, 1 Middle turbinate.	Median OS: 80 months, 1-year OS:82.6%, 5-year OS:54.5%.	15 S, 2 RT, 1 palliative chemotherapy
Lundberg et al, 2019. [31]	Finland	Single center	1983-2016	median 16, mean 38 (range 1-229)	58	Retrospective cohort study	SNMM	median 75 (range 43-95)	53	16 Nasal cavity, 9 Septum, 3 Nasal floor, 15 Nasal lateral wall, 2 Vestibule, 9 Maxillary sinus, 4 Ethmoid sinus.	5-year DSS: 27%, 5-year OS: 25%.	44 curative intent: 30 S only, 13 S + RT, 1 RT only; 14 palliative intent: 11 surgical debulking or radiofrequency ablation, 1 RT only, 2 systemic therapy
Letievant et al, 2016. [32]	France	Single center	1994-2014	median 43	14	Retrospective cohort study	SNMM	median 67	43	Primary site: 8 Nasal cavities and 6 Paranasal sinuses: 5 Nasal cavity, 3 Inferior turbinate, 4 Maxillary sinus, 2 Ethmoidal sinus	Median recurrence-free interval: 28.7 months, 2-year OS: 43% and 5-year OS: 32%.	All S (4 S only, 10 adjuvant external RT)
Khan et al, 2014. [33]	US	Database: SEER	1973-2009	NR	567	Retrospective cohort study	SNMM, Nasal cavity, Paranasal sinuses	Nasal cavity mean 69.01, Maxillary sinus mean 69.01	56	Primary site: 383 Nasal cavity, 96 Maxillary sinus, 41 Ethmoid sinus, 11 Overlapping lesion of accessory sinuses.	Overall 1-year and 5-year DSS: 76.09% and 31.49%. Overall relative survival (the ratio of the observed survival rate to the expected age-adjusted background survival rate): 75.00% at 1 year and 31.40% at 5 years. 5-year DSS: 36.66% for patients diagnosed with nasal cavity disease, 23.80% for patients with maxillary sinus tumors, and 18.20%	Maxillary sinus involvement: 76.34% RT; nasal cavity involvement: 52.28% RT

											for patients with ethmoid sinus disease.	
Wang et al, 2020. [34]	China	Single center	2008.1-2017.12	median 22 (range 4-96)	32	Retrospective cohort study	SNMM	mean 67.4 (SD10.8)	47	Primary site: 23 (14/9) Nasal cavity (inferior turbinate/others), 10 Maxillary sinus, 3 Ethmoidal sinus.	1-, 3-, and 5-year OS is 80.6%, 36.1%, and 13.9%.	20 S only, 13 S + RT, 3 RT only
Dréno et al, 2017. [35]	France	Single center	1988-2015	mean 50	44	Retrospective cohort study	SNMM	mean 71.2 (range 50-96)	59	Primary site: 29 Nasal cavities, 15 Sinus (9 Maxillary, 1 Frontal, 5 Ethmoid); Local extension: 15 Nasal cavities, 13 Sinuses, 4 Ethmoid cribriform plate, 2 Anterior skull base, 3 Nasopharynx, 1 Infratemporal fossa, 2 Lacrimal ducts, 3 Orbital floor/orbit, 1 Cavernous sinus; Metastatic extension: 2 Bone, 1 Skin, 2 Multiple metastasis.	OS: 71.5% in 1 year and 33% in 5 years.	42 S, 19 external RT, 8 chemotherapy, 14 immunotherapy
Vandenhende et al, 2012. [36]	France	Single center	1991.1-2008.7	mean 31.3 (range 2-99)	25	Retrospective cohort study	SNMM	median 68 (range 29-90)	52	11 Lateral nasal wall (nasal turbinates, intersinonasal septum, or ethmoid sinus), 4 Maxillary sinus, 5 Nasal septum, 1 Floor of the nasal fossa, 4 Outside the nasal fossa and sinuses. Data for 17 patients were analyzed：11 Nasal fossa, 5 Sinus.	Data for 17 patients were analyzed：Nasal fossa：3-year OS 61.4%, 3-year DFS 40.9%; Sinus: 3-year OS 33.3%. 3-year DFS: 0.	23 S, 15 PORT
Liétin et al, 2010. [37]	France	Multicenter: France	1998-2008	mean 36.3 (range 2-96)	10	Retrospective cohort study	primary SNMM	mean 71 (range 61-85)	70	Primary site and extension: 6 Nasal cavity, 1 Middle meatus with ethmoid bone extension, 1 Middle meatus and maxillary sinus with posterior extension, 1 Middle meatus with fronto-ethmoido-maxillary extension, 1 All sinuses.	5-year OS: 40%.	4 transnasal approach, 1 transnasal approach + RT, 3 PLN + RT, 1 degloving + RT, 1 paralateronasal
Rojas-Lechuga et al, 2022. [38]	Spain	Multicenter: Spain	1984.7-2020.7	median 39.6	50	Retrospective cohort study	SNMM	mean 70.4 (SD 12.5, range 40-95)	52	Primary site: 24 Nasal cavity, 26 Paranasal sinuses.	3-year MSS: 51.2%, 5-year MSS: 29.5%.	21 S, 20 S + RT, 9 palliative

Amit et al, 2017. [5]	US	Single center	1991-2016	median 28 (range 2-220)	152	Retrospective cohort study	SNMM, primary site in sinonasal cavity, surgery	median 64, mean 63 (SD 13, range 34-91)	57	Primary site: 119 Nasal cavity, 32 Paranasal sinus, 1 Cannot be determined.	Five-year OS and DSS were 41 and 61%; Five-year OS: 39, 42, 47, and 27% for the surgery only, PORT, POCRT, and neoadjuvant chemotherapy groups.	57 S only, 81 PORT, 14 neoadjuvant chemotherapy (cisplatin, vinblastine, and dacarbazine) + PORT
Sun et al, 2014. [39]	China	Single center	1976.1-2005.12	NR	65	Retrospective cohort study	SNMM with treatment	median 55 (range 2-79)	29	Primary site: 52 Nasal cavity, 13 Paranasal sinus.	3-year OS: 36.5%, 5-year OS: 29.7%; median survival time was 24 months (range, 1-264 months).	51 S, 20 RT
Dauer et al, 2008. [40]	US	Single center	1955-2003	for 4 patients alive with no evidence of disease: 37-196	61	Retrospective cohort study	SNMM with primary treatment in the institution	mean 67 (range 30-91)	49	NR	The cancer-specific survival rate was 48.9% and 22.1% at 3 and 5 years.	29 wide local excision alone, 17 excision + RT, 10 primary RT alone, 5 radium implants and chemotherapy
Ajmani et al, 2017. [41]	US	Database: NCDB	2004-2013	NR	696	Retrospective cohort study	SNMM treated with primary surgery, M0	48.6%<70, 51.4%≥70.	54	Primary site: 525 Nasal cavity, 179 Paranasal sinus.	5-year OS: 24%. Median survival was 25.0 months for those treated with surgery alone, compared to 28.3 months for those receiving adjuvant RT.	305 S alone, 399 adjuvant RT
Zhu et al, 2024. [42]	China	Database: SEER	2000-2019	median 18 (IQR: 8-39)	459	Retrospective cohort study	SNMM, Nasal cavity, Paranasal sinuse	28.5% < 65, 41.0% 65-79, 30.5% ≥80	56	Primary site: 366 Nasal cavity, 93 Paranasal sinus.	The median OS in the cohort from the SEER database was 28 months. The 1-year, 3-year and 5-year OS rates were 69.8%, 40.4%, and 30.0%.	383 S, 76 no S, 295 RT, 164 no RT, 37 chemotherapy, 422 no chemotherapy.
Yu et al, 2015. [43]	China	Single center	1999.10-2013.6	median 70.8 (5-135)	29	Retrospective cohort study	Nasal mucosa malignant melanoma,	mean 61.5 (range 49-73)	38	Primary site: 7 Nasal septum, 22 Lateral wall of nasal cavity/paranasal sinus.	3-year OS: 48.3%, 5-year OS: 27.6%.	22 S, 11 RT ≤ 54 Gy, 17 RT > 54 Gy, 17 chemotherapy

received treatment

* SNMM, sinonasal mucosal melanoma; MM, mucosal melanoma; HNMM, head and neck mucosal melanoma; OS, overall survival; DSS, disease-specific survival; DFS, disease-free survival; PFS, progression-free survival; MSS, melanoma-specific survival; RFS, recurrence-free survival; LR, local recurrence; RR, regional recurrence; DM, distant metastasis; RS, relative survival; S, surgery; CFR, craniofacial resection; Gy, Gray; PORT, postoperative radiotherapy; POCRT, postoperative chemoradiotherapy; RT, radiotherapy; SD, standard deviation; IQR, interquartile range; NR, not reported; NCDB, National Cancer Database; SEER, Surveillance, Epidemiology, and End Results (database); NCR, Netherlands Cancer Registry; REFCOR, Réseau d'Expertise Français sur les Cancers ORL Rares (French rare head and neck cancer network); NOS, not otherwise specified; #: Studies marked with '#' were included in the meta-analysis.
